# Overexpression of *AmCML24* Improves Abiotic Stress Tolerance and Upregulates Stress-Responsive Genes in Arabidopsis

**DOI:** 10.3390/plants15030420

**Published:** 2026-01-30

**Authors:** Jing Niu, Jiexia Bai, Shijing Sun, Yuting Fan, Jiaxin Li, Yuhan Cao, Lin Li, Haoyuan Jin, Lili Zhang, Fanjuan Meng, Qiuxiang Luo

**Affiliations:** 1 Key Laboratory of Saline-Alkali Vegetation Ecology Restoration, Ministry of Education, College of Life Sciences, Northeast Forestry University, Harbin 150040, China; 2 College of Agriculture, Northeast Agricultural University, Harbin 150030, China; 3 Jilin Provincial Key Laboratory of Tree and Grass Genetics and Breeding, College of Forestry and Grassland Science, Jilin Agricultural University, Changchun 130118, China

**Keywords:** abiotic stress, *AmCML24* gene, molecular mechanism

## Abstract

With the intensification of global climate change, environmental issues such as soil salinization and drought have exerted an increasingly prominent impact on plants. Tibetan peach (*Amygdalus mira*), a rare native tree species of the genus Amygdalus in the Rosaceae family, possesses extremely strong tolerance to cold, drought, and disease stress. In the previous study, we found that the protein abundance of a calcium-binding protein, AmCML24, was significantly upregulated in Tibetan peach under drought stress, leading us to hypothesize that it plays an important role in the molecular mechanisms underlying plant responses to abiotic stresses. Therefore, this study focuses on AmCML24, aiming to preliminarily characterize the stress-tolerant function of AmCML24 and explore its biological role in plant tolerance to saline–alkali and drought stresses. Results demonstrated that *AmCML24* responds to multiple abiotic stresses. Yeast and *Arabidopsis thaliana* lines overexpressing *AmCML24* exhibited enhanced tolerance to NaCl, NaHCO_3_, and mannitol stresses, with a significant upregulation in the expression of stress-responsive genes. This study lays a solid foundation for deciphering the stress regulatory network of Tibetan peach and elucidating the biological function of *AmCML24*, while also providing a scientific basis for the genetic improvement, exploitation, and utilization of Tibetan peach germplasm resources.

## 1. Introduction

As a unique wild fruit tree species of the genus *Amygdalus* (Rosaceae) native to China, Tibetan peach (*Amygdalus mira*) is one of the most widely distributed wild fruit tree germplasms in Tibet. It is also a rare “living fossil group” germplasm both domestically and internationally [1]. This species not only exhibits prominent stress tolerance traits such as cold tolerance, drought tolerance, barren tolerance, and disease tolerance but also holds high economic value [2], i.e., its fruits are nutrient-rich; its kernels can be pressed for oil with a high content of unsaturated fatty acids; and the kernels have been used as a medicinal material in the Tibetan medicine system for over 1300 years [3]. Additionally, it possesses ornamental value and ecological functions of water conservation and soil consolidation, and plays a key biological role in maintaining species diversity and breeding new *Amygdalus* varieties. However, affected by climate change and human activities, the wild resources of Tibetan peach have become endangered and have been included in the list of key protected plants [4,5,6]. Therefore, elucidating its stress tolerance mechanisms is crucial for the conservation, exploration, and utilization of this germplasm resource.

The aggravation of global climate change has exacerbated the constraints of abiotic stresses on plant growth, among which saline–alkali stress and drought stress are particularly harmful. Globally, more than 900 million hectares of land are threatened by salinization, with saline–alkali land in China covering an area of 100 million hectares. On the other hand, drought is the primary factor limiting plant productivity [7,8]. These stresses inhibit plant growth through multiple pathways. Saline–alkali stress induces ion toxicity, such as high-concentration Na^+^ interfering with nutrient absorption, along with osmotic imbalance, which result in cell dehydration and oxidative damage where the accumulation of reactive oxygen species (ROS) destroys cell membranes [9,10,11]. Moreover, alkali stress, due to its high pH value, damages root structure and cellular pH homeostasis, resulting in more severe harm than salt stress of the same concentration [12,13]. Drought stress reduces cell turgor and inhibits photosynthesis (e.g., stomatal closure leads to insufficient CO_2_ supply), and also induces excessive ROS production, which damages photosynthetic apparatus and biological macromolecules [14,15]. To cope with these stresses, plants have evolved a series of adaptive mechanisms, including the accumulation of osmoregulatory substances (such as proline) [16], the activation of antioxidant enzyme systems (such as SOD and CAT) [17], and the regulation of hormone signaling networks mediated by abscisic acid (ABA) [18].

Calcium signaling is a core regulatory pathway for plants to respond to environmental stresses and regulate growth and development, while calcium-binding proteins are key carriers for calcium signal transduction [19]. The plant calcium-binding protein family includes calmodulin (CaM), calmodulin-like proteins (CMLs), and calcineurin B-like proteins (CBLs), among which CMLs are unique to plants [20]. Previous studies have confirmed that *CML* genes, such as *AtCML8* in *Arabidopsis thaliana* and *OsMSR2* in *Oryza sativa*, play important roles in responding to salt and drought stresses, and can enhance plant stress tolerance by regulating the expression of stress-related genes [21,22].

In our previous proteomic study on Tibetan peach under drought stress, it was revealed that the protein abundance of a calcium-binding protein was significantly upregulated after treatment [23]. Based on this finding, it was hypothesized that this protein is closely associated with the stress tolerance of Tibetan peach. Subsequently, partial sequences of this protein were obtained via mass spectrometry identification, and its corresponding encoding gene was cloned from the transcriptome of Tibetan peach. Sequence alignment analysis revealed that this gene shared high homology with *AtCML24* of *Arabidopsis thaliana*. In accordance with the nomenclature rules for homologous plant genes, this gene was designated as *AmCML24.*

To address the knowledge gap regarding the functional roles of AmCML24 proteins from Tibetan peach in abiotic stress adaptation, the present study aims to systematically characterize the biological function of AmCML24 in mediating plant stress tolerance. The central hypothesis of this research is that AmCML24 encodes a functional calcium-binding protein that enhances plant tolerance to salt, alkali, and drought stresses by regulating the transcription of downstream stress-responsive genes in heterologous expression systems (yeast and Arabidopsis). The overall goal of this work is to systematically characterize the biological function of *AmCML24* in mediating plant abiotic stress tolerance and to preliminarily explore its underlying regulatory mechanism. To achieve this goal, three specific research objectives were established as follows: (1) to determine the subcellular localization of the AmCML24 protein using a GFP fusion transient expression system in onion bulb mesophyll epidermal cells; (2) to evaluate the functional role of *AmCML24* in improving abiotic stress tolerance via heterologous overexpression assays in yeast and Arabidopsis under NaCl, NaHCO_3_, and mannitol treatments; (3) to identify the regulatory effects of *AmCML24* overexpression on the expression profiles of key stress-responsive marker genes in Arabidopsis. The following sections describe the materials and methods employed to test these hypotheses and objectives, followed by the presentation and discussion of the resulting data.

## 2. Results

### 2.1. Cloning, Expression Analysis, and Subcellular Localization of AmCML24 from Tibetan Peach

**Figure 1:**
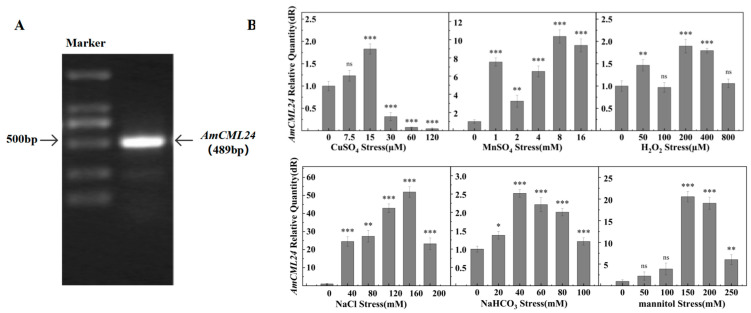
(**A**) Cloning of the AmCML24 gene. Note: Marker indicates DL2000 DNA marker (band sizes: 2000 bp, 1000 bp, 750 bp, 500 bp, 250 bp, 100 bp). (**B**) RT-qPCR analysis was performed to detect the AmCML24 gene expression profiles of 3-month-old Tibetan peach subjected to different stress treatments over a continuous 7-day period. For RT-qPCR analysis, 18S rRNA was used as an endogenous control, the error bar is the standard error of 3 replicates (* *p* < 0.05, ** *p* < 0.01, *** *p* < 0.001, ns: not significant). Dunnett’s multiple comparisons test.

**Figure A1:**
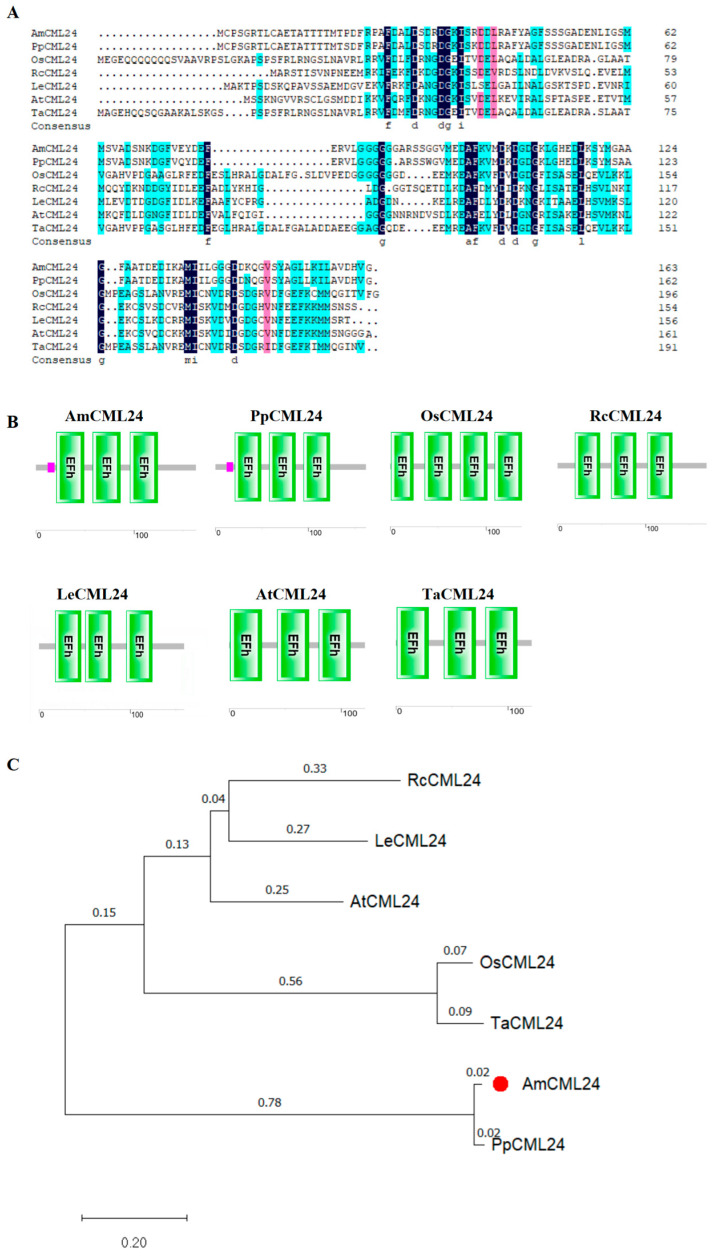
Sequence and motif analyses of AmCML24 and homologous CML proteins. (**A**) Multiple sequence alignment of AmCML24 and CML24 orthologs from representative plant species. (**B**) Schematic representation of the conserved domain architecture of CML24 proteins from diverse plant species. The green boxes represent EF-hand calcium-binding domains. The scale bar at the bottom represents the relative length of the protein sequence in amino acids. (**C**) Phylogenetic tree of AmCML24 and its orthologs constructed using the neighbor-joining method.

To investigate the potential function of the *AmCML24* gene from Tibetan peach in response to abiotic stresses, and to clarify its gene sequence characteristics and stress response patterns, we first cloned the *AmCML24* gene using cDNA from Tibetan peach leaves as a template. The open reading frame (ORF) of this gene is 489 bp in full length (Figure 1A). After successful cloning and sequencing of the target fragment, we further performed a series of bioinformatics analyses on the AmCML24 protein, including multiple sequence alignment of amino acid sequences and prediction of protein structural domains, with detailed results presented in Appendix A Figure A1. The results showed that AmCML24 contains three conserved EF-hand calcium-binding domains, which share high homology with the functional domains of CML24 proteins from other plant species, and exhibits the closest genetic relationship with *Prunus persica* (peach). Further, with Tibetan peach cDNA as the template, the expression characteristics of the *AmCML24* gene under different abiotic stress treatments were detected via quantitative real-time polymerase chain reaction (RT-qPCR).

RT-qPCR analysis revealed that *AmCML24* exhibited a differential expression pattern under various stresses. As shown in Figure 1B, under NaCl stress, the expression level reached its peak when treated with 160 mM NaCl, which was significantly upregulated by 50-fold compared with the control, followed by a gradual slowdown in the increasing amplitude; under NaHCO_3_ stress, the expression level increased by 2.5-fold under 40 mM treatment, while the response gradually weakened under high-concentration treatments; under mannitol stress, the expression level surged by 20-fold when treated with 150 mM mannitol, and the increasing amplitude decreased at subsequent concentrations; under heavy metal stress, the expression level increased by 1.8-fold under 15 μM CuSO_4_ treatment, whereas high concentrations inhibited its expression; the expression level was significantly upregulated by 10-fold under 8 mM MnSO_4_ treatment; under H_2_O_2_ stress, the expression level increased by 2-fold under 200 μM treatment, while no significant changes were observed at other concentrations.

The above results indicate that *AmCML24* responds to varying degrees under NaCl, NaHCO_3_, mannitol, CuSO_4_, MnSO_4_, and H_2_O_2_ stresses. Among these, the response to NaCl, NaHCO_3_, and mannitol stresses was the most significant. Therefore, all subsequent experiments were conducted under these three stress conditions.

**Figure 2:**
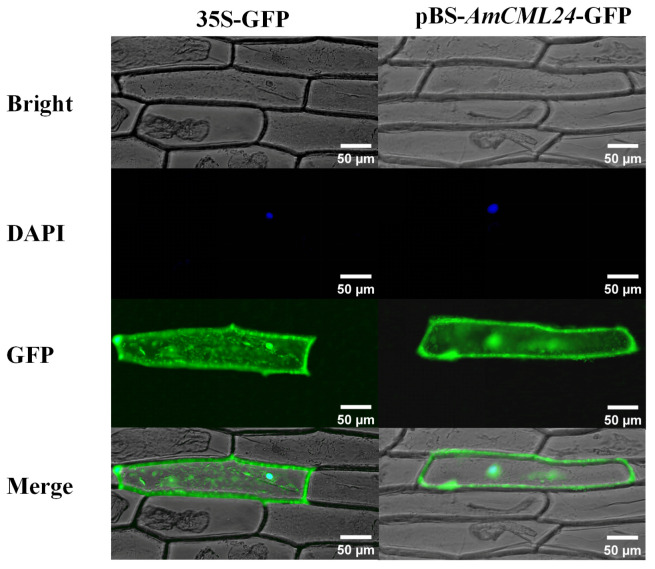
Subcellular localization of pBS-*AmCML24*-GFP. This figure shows the AmCML24 fusion protein labeled with green fluorescent protein (35S-GFP), together with the corresponding 35S-GFP fluorescence field, DAPI fluorescence field, and merged image.

Subsequently, the gene gun-mediated bombardment method was used to transform onion epidermal cells, and it was found that AmCML24 is localized in the cytoplasm and nucleus of onion epidermal cells (Figure 2).

### 2.2. Overexpression of AmCML24 Enhances Yeast Tolerance to NaCl, NaHCO_3_, and Mannitol

To clarify whether the *AmCML24* gene exerts an effect on the ability of yeast to resist abiotic stresses, we systematically investigated the functional role of this gene through tolerance analysis experiments under liquid culture conditions.

**Figure 3:**
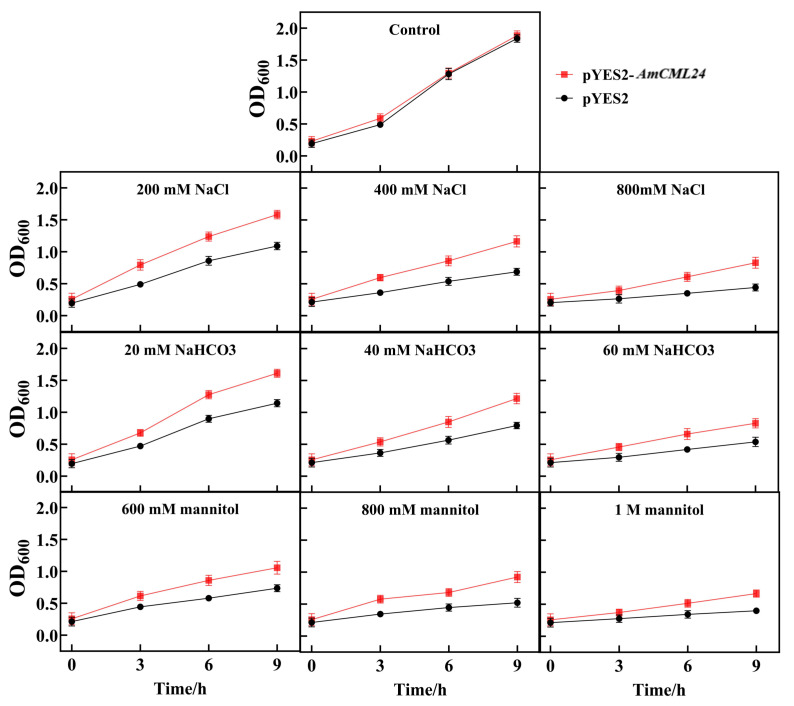
Effect of *AmCML24* expression on yeast growth under NaCl, NaHCO_3_, and mannitol stresses. The two yeast strains were separately resuspended in 1 mL of YPD liquid medium supplemented with different stressors, followed by shaking incubation at 28 °C. Three biological replicates were set up for each group. Samples were collected at 3, 6, and 9 h, and the OD_600_ values were measured.

As shown in Figure 3, under stress-free conditions, there was no significant difference in the growth curves between the two groups of yeast. With the increase in the concentration of different abiotic stresses, the growth of yeast was inhibited; however, under different stress concentrations, the growth rate of yeast transformed with pYES2-*AmCML24* was consistently higher than that of the empty vector control yeast. Particularly, under high stress concentrations, such as 800 mM NaCl, 60 mM NaHCO_3_, and 1 M mannitol stresses, the growth rate of yeast transformed with pYES2-*AmCML24* was much higher than that of the empty vector control yeast. The results of this liquid cultivation experiment confirmed that, under gradient stress conditions, the yeast strain overexpressing *AmCML24* exhibited a higher survival rate compared with the empty vector control, demonstrating that the overexpression of *AmCML24* can effectively enhance the tolerance of yeast to NaCl, NaHCO_3_, and mannitol stresses.

### 2.3. The Overexpression of the AmCML24 Gene in Arabidopsis thaliana Enhances Its Tolerance to Saline–Alkali and Drought Stresses

**Figure 4:**
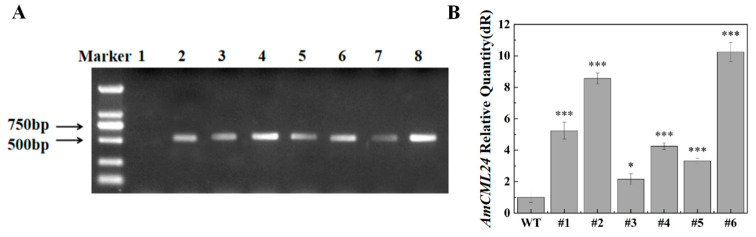
Screening of transgenic Arabidopsis positive lines. (**A**) PCR identification of transgenic *Arabidopsis thaliana*; lines 1–8 indicate: 1-negative control, 2-positive control, 3-#1, 4-#2, 5-#3, 6-#4, 7-#5, 8-#6. Note: Marker indicates DL2000 DNA marker (band sizes: 2000 bp, 1000 bp, 750 bp, 500 bp, 250 bp, 100 bp). (**B**) *AmCML24* relative expression in wild-type and transgenic lines. The relative expression level was normalized to the wild-type expression level, which is considered as 1.0; the 18S rRNA was used as an internal control. The error bar is the standard error of 3 replicates (* *p* < 0.05, *** *p* < 0.001). Dunnett’s multiple comparisons test.

To explore the role of the *AmCML24* gene in salt–alkali and drought tolerance, we transformed it via the Agrobacterium-mediated method into the model plant *Arabidopsis thaliana* via heterologous expression, and verified its upregulation effect through RT-qPCR analysis. In the overexpression lines, the expression level of the *AmCML24* gene was significantly increased, among which the relative expression levels of the #1, #2, and #6 lines were more prominently upregulated compared with the wild type (Figure 4); these were identified as high-expression candidate lines, hereinafter referred to as #1, #2, and #3. This indicates that the *AmCML24* overexpression lines were successfully constructed.

**Figure A2:**
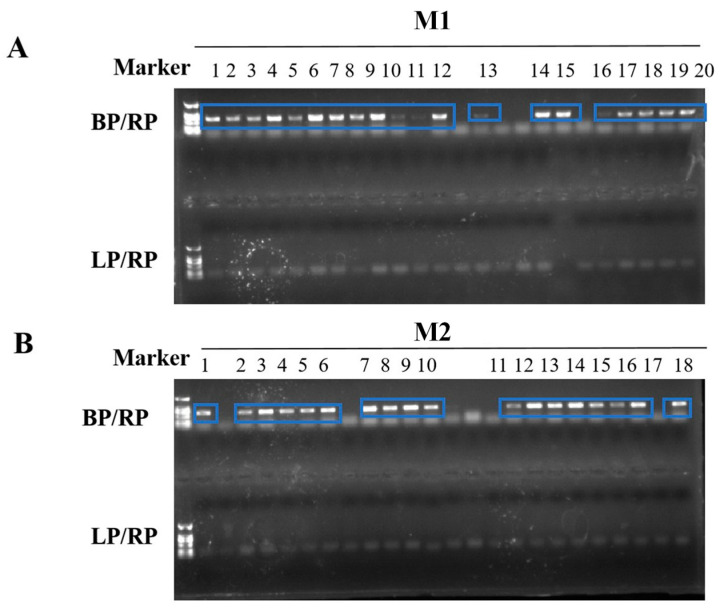
Identification of homozygous mutants in Arabidopsis thaliana. M: DL2000 DNA marker; LP/RP: wild-type allele-specific primer pair; RP/BP: mutant allele-specific primer pair. Homozygous mutant lines only amplify the mutant band corresponding to the RP/BP primer pair.

To further verify the function of the *AmCML24* gene in salt–alkali and drought of *Arabidopsis thaliana*, T-DNA insertion mutants SALK_079293C (M1) and SALK_058127C (M2) were purchased in this study based on the sequence information of the *AmCML24* gene from Tibetan peach. Homozygous mutant lines were successfully identified via the three-primer method, and the corresponding identification profile is provided in Figure A2.

**Figure 5:**
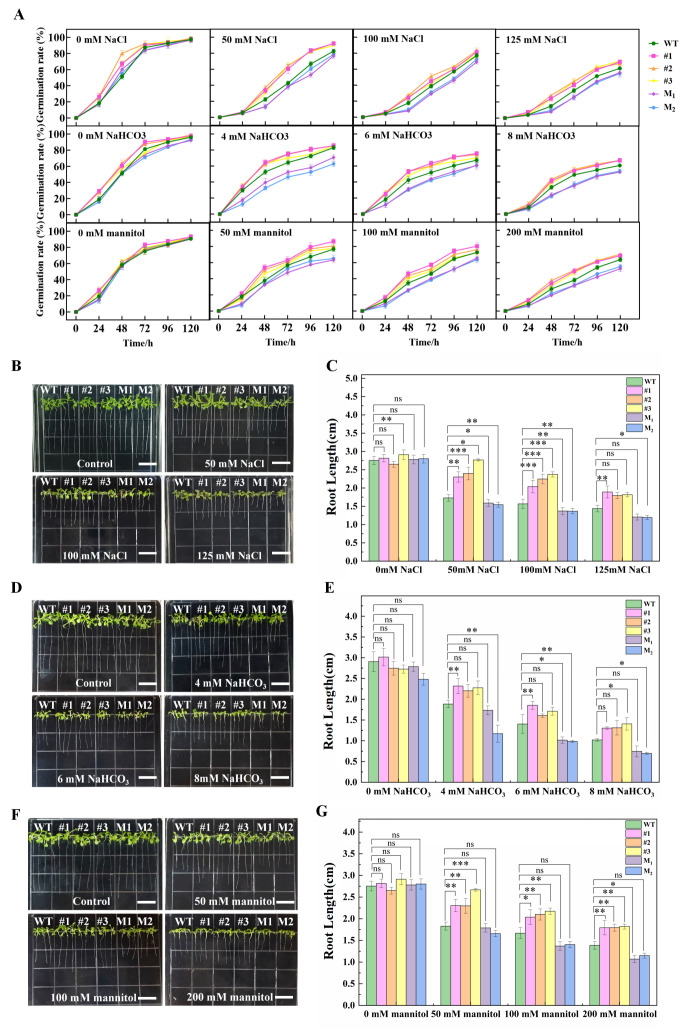
(**A**) Germination analysis of transgenic *Arabidopsis thaliana* under saline–alkali and drought stresses. Three biological replicates were established for each treatment, with 60 seeds per replicate (n = 60). (**B**–**G**) Root length analysis of transgenic *Arabidopsis thaliana* under saline–alkali and drought stresses. Seedlings with uniform taproot lengths were selected and transferred to 1/2 MS treatment plates supplemented with different gradient stressors; after 7 days of treatment, root system images were recorded (scale bar = 1 cm), and root length measurement and analysis were performed using Image J ImageJ Version 1.54r software; the error bar is the standard error of 3 replications (* *p* < 0.05, ** *p* < 0.01, *** *p* < 0.001). Dunnett’s multiple comparisons test.

First, seed germination and root length measurements were performed on the wild type (WT), *AmCML24*-overexpressing transgenic lines, and the mutant lines M1 and M2. The results demonstrated that, under normal conditions, the germination rates of all Arabidopsis lines were basically consistent. Under salt–alkali and drought stress, the seed germination rates of the three types of lines all decreased with the increase in stress concentration. Notably, the germination rates of the WT, M1, and M2 were consistently lower than that of the *AmCML24*-overexpressing transgenic Arabidopsis, with the germination rates of the mutants being the lowest. This suggests that the *AmCML24*-overexpressing transgenic Arabidopsis exhibits stronger salt, alkali, and drought tolerance during the germination stage. Meanwhile, the same trend was observed in the root length measurements (Figure 5).

**Figure 6:**
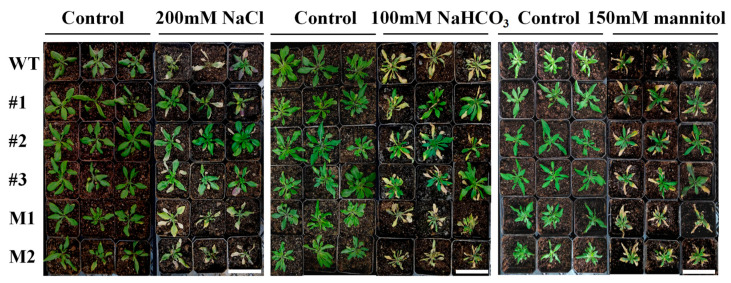
Effects of saline–alkali and drought stress on the growth phenotype of WT and *AmCML24*-overexpressing transgenic Arabidopsis seedlings. Three-week-old WT, transgenic, and mutant *Arabidopsis thaliana* plants were separately cultured under normal conditions, 200 mM NaCl, 100 mM NaHCO_3_, and 150 mM mannitol conditions for 14 days, followed by phenotypic comparison and analysis. Three biological replicates were set up for each plant line. Scale bar = 10 cm.

Subsequently, three-week-old *AmCML24* overexpression lines, WT lines, and mutant lines were cultured under normal growth conditions, salt–alkali stress, and drought stress for 14 days, respectively, and their phenotypes were compared. The results (Figure 6) showed that, under normal growth conditions, there was no significant difference in the growth status of all tested plants. Under stress conditions, however, the leaves of wild-type plants showed wilting, yellowing, and curling; in contrast, most overexpression lines could still grow normally with leaves remaining green and fully expanded, while the mutants were severely inhibited, and some even died.

To clarify the salt–alkali and drought tolerance mechanism mediated by *AmCML24* at the physiological level, the contents of soluble proteins, malondialdehyde (MDA), and proline (Pro) were determined in this study. The results revealed that the variation trends of soluble protein, MDA, and Pro contents in the *AmCML24* overexpression lines were consistent with the performance of the transgenic Arabidopsis in stress tolerance. Under salt–alkali and drought stress conditions, the contents of soluble proteins, MDA, and Pro in WT plants were significantly higher than those in the overexpression plants.

**Figure 7:**
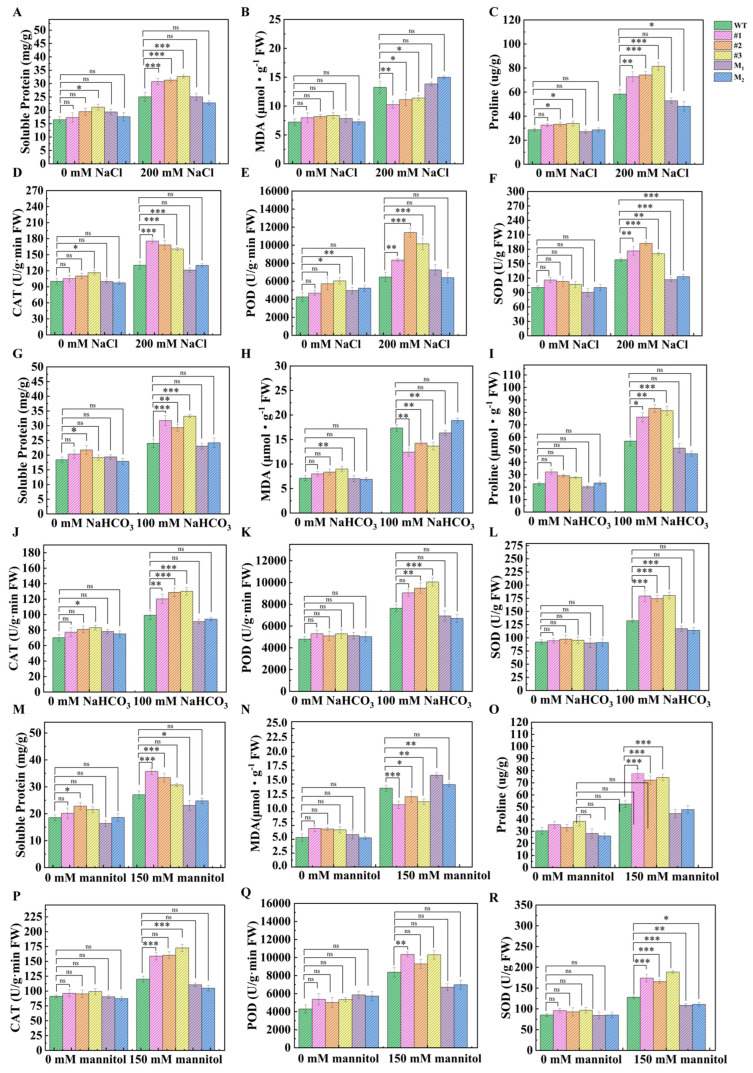
Effects of saline–alkali stress and drought stress on the physiology of WT, *AmCML24*-overexpressing transgenic, and mutant Arabidopsis. The determination of soluble protein, MDA, Pro, CAT, POD, and SOD contents in various Arabidopsis lines under NaCl stress (**A**–**F**), NaHCO_3_ stress (**G**–**L**), and mannitol stress (**M**–**R**). For each plant line under each treatment, 3 independent biological replicates were set up, with each biological replicate consisting of 3 individual plants. The error bar is the standard error of 3 replications (* *p* < 0.05, ** *p* < 0.01, *** *p* < 0.001). Dunnett’s multiple comparisons test.

To investigate whether the reduction in reactive oxygen species (ROS) content and the alleviation of lipid peroxidation were caused by changes in antioxidant enzyme activity, the activities of catalase (CAT), superoxide dismutase (SOD), and peroxidase (POD) were further analyzed. Under non-salt–alkali and non-drought stress conditions, there was no significant difference in the activities of CAT, SOD, and POD between the transgenic Arabidopsis and WT plants. Under salt–alkali and drought stress conditions, however, the activities of CAT, SOD, and POD in all tested lines increased, and the activities of these enzymes in the overexpression plants were significantly higher than those in the WT plants (Figure 7).

In addition, this study also analyzed the transcript levels of genes related to salt–alkali and drought stress responses via RT-qPCR, with three biological replicates and three technical replicates performed for each assay. The detected genes included salt stress response genes (*AtCML8*, *AtSOS1*), alkali stress response genes (*AtMYB44*, *AtSOS1*), drought stress response genes (*AtHB7*, *AtABF2*), and abscisic acid (ABA) pathway-related genes (*AtNCED3*, *AtABI4*, *AtMYB71*).

**Figure 8:**
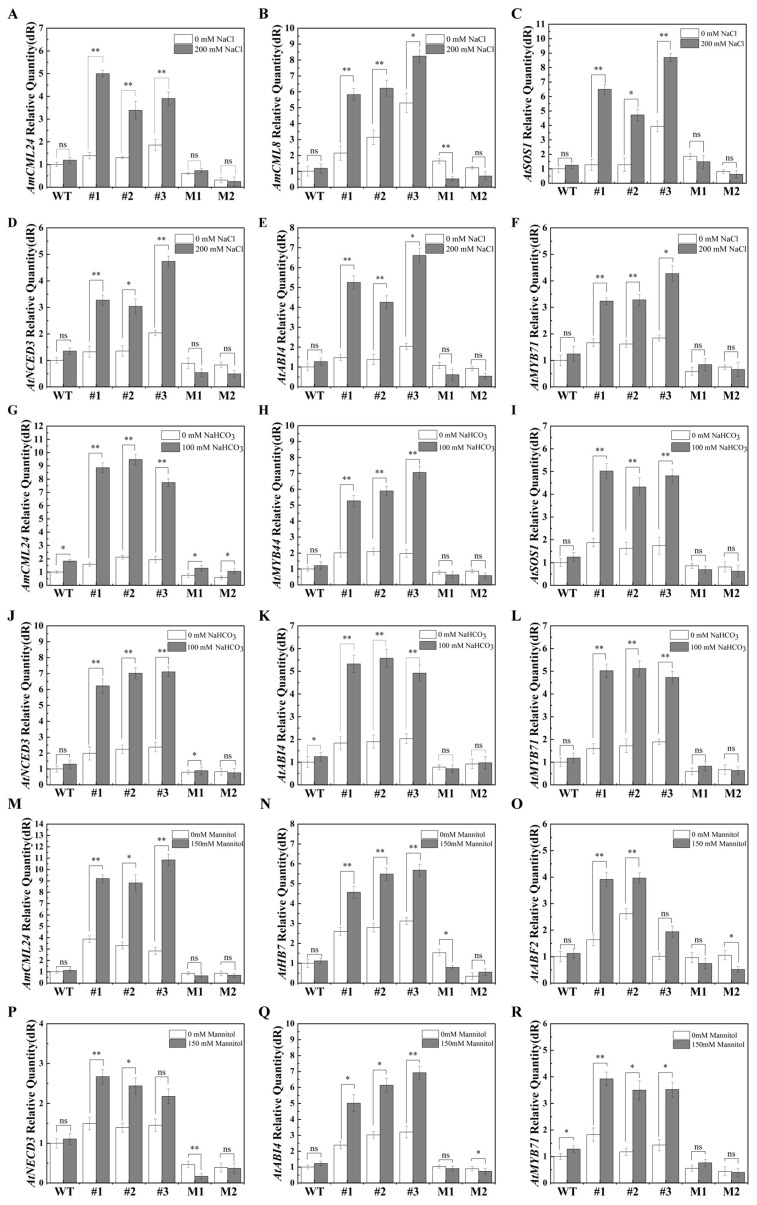
Effects of abiotic stress on the expression of stress-related genes. Analysis of relative expression levels of related genes under 200 mM NaCl stress (**A**–**F**), 100 mM NaHCO_3_ stress (**G**–**L**), and 150 mM mannitol stress (**M**–**R**). The error bar is the standard error of three replicates (* *p* < 0.05, ** *p* < 0.01). Student’s *t*-test.

These data showed that, compared with WT plants, *AmCML24*-overexpressing transgenic Arabidopsis lines exhibited a significant upregulation of stress-responsive genes under adverse stress conditions. Specifically, under salt–alkali stress, the expression level of *AtSOS1*—a core regulatory gene in the SOS pathway that mediates cellular ion homeostasis—was notably upregulated in the *AmCML24*-overexpressing transgenic Arabidopsis. Consistently, the expression levels of key ABA signaling pathway genes (*AtNCED3*, *AtABI4*, *AtMYB71*) also increased significantly in the overexpression lines relative to the WT controls (Figure 8).

## 3. Discussion

### 3.1. Cloning, Subcellular Localization, and Expression Response Analysis of AmCML24 Gene from Tibetan Peach Under Abiotic Stresses

A large body of research has confirmed that nearly all extracellular stimuli can induce the generation of Ca^2+^ signals in plants, including light, touch, gravity, phytohormones, and various biotic and abiotic stresses. Calcium-binding proteins are key proteins in plants that transmit cellular Ca^2+^ signals and respond to hormones, environmental changes, and stresses. Accompanied by Ca^2+^ signal transduction, calcium-binding proteins are involved in a variety of physiological reactions and activities in plants, including stress tolerance, cell differentiation, and stomatal movement [24]. In this study, *AmCML24* was cloned from the cDNA library of Tibetan peach. The gene gun method was used to bombard onion epidermal cells for subcellular localization analysis of AmCML24, and the results showed that AmCML24 was localized to the cytoplasm and nucleus of onion epidermal cells. During plant growth and development, plants are often affected by various stress factors, which significantly inhibit plant growth and development and cause crop yield reduction [25]. Currently, calcium-binding proteins have been identified in multiple plant species, such as *Arabidopsis thaliana* [26], rice (*Oryza sativa*) [27], and soybean (*Glycine max*) [28]; they play important roles under different stress conditions. In this study, Tibetan peach seedlings were treated with different abiotic stresses (NaCl, NaHCO_3_, mannitol, CuSO_4_, MnSO_4_, and H_2_O_2_). It was found that the expression level of *AmCML24* changed significantly under NaCl, NaHCO_3_, and mannitol stresses, indicating that *AmCML24* responds to NaCl, NaHCO_3_, and mannitol stresses. This finding provides a research direction and theoretical basis for subsequent studies on the response of *AmCML24* to abiotic stresses.

### 3.2. Functional Verification of AmCML24 in Stress Tolerance in Yeast

The molecular mechanisms underlying the responses of yeast and plants to adverse environments are highly conserved during evolution, which provides an important theoretical basis for verifying the functions of plant stress-resistant genes using the yeast expression system [29]. For the study of a specific gene, the yeast expression system is of great help in exploring its stress tolerance function. As early as the 1990s, researchers used the yeast expression system to study the salt tolerance of plants [30]. Subsequent numerous studies have further confirmed that this system exhibits significant value in elucidating the functions of various stress-responsive genes in plants, such as drought tolerance, saline–alkali tolerance, and heavy metal tolerance, thus building a critical bridge for subsequent in-depth functional verification in plant tissues.

Following this well-established research strategy, the present study conducted a comparative analysis between the pYES2-*AmCML24* recombinant strain and the empty vector control strain. The results showed that the growth rate of the recombinant strain was significantly higher than that of the empty vector yeast strain in liquid stress media containing NaCl, NaHCO_3_, and mannitol. This phenotypic difference directly indicates that heterologous expression of the *AmCML24* gene can endow yeast cells with tolerance to multiple stress factors, thus preliminarily verifying the potential broad-spectrum stress-resistant function of this gene. Furthermore, these experimental results are consistent with the previously described prominent expression response characteristics of the *AmCML24* gene to NaCl, NaHCO_3_, and mannitol stress in Tibetan peach seedlings. These findings further corroborate the core role of the *AmCML24* gene in plant stress responses, and provide robust preliminary evidence for subsequent in planta verification of its stress-resistant function and dissection of the underlying molecular mechanisms.

### 3.3. Overexpression of AmCML24 Improves Stress-Resistant Phenotypes and Physiological Adaptability in Transgenic Arabidopsis thaliana

In this study, homozygous T3-generation *AmCML24*-overexpressing transgenic *Arabidopsis thaliana* lines were successfully developed. Through stress treatment experiments with salt (NaCl), alkali (NaHCO_3_), and drought (simulated by mannitol), the regulatory effects of this gene on the stress-resistant phenotypes and physiological characteristics of plants were systematically investigated. The detection results of phenotypic and physiological indicators under stress conditions showed that overexpression of *AmCML24* significantly improved the germination rate and root elongation of transgenic *Arabidopsis thaliana*, and the seedlings also exhibited obvious stress-resistant phenotypic advantages, which was highly consistent with the stress-resistant functional characteristics of genes derived from Tibetan peach.

In-depth analysis of the physiological mechanism showed that, compared with the WT and mutant lines, the contents of soluble proteins and proline in the leaves of the transgenic lines were significantly increased, while the accumulation of MDA was significantly lower. Meanwhile, the activities of core antioxidant enzymes such as CAT, POD, and SOD were significantly enhanced. Previous studies have confirmed that proline and soluble proteins, as important osmotic adjustment substances, can enhance the osmotic regulation ability of plant cells and alleviate cell dehydration damage under stress when their contents are increased [31,32]. In contrast, MDA is a key product of membrane lipid peroxidation, and the decrease in its accumulation is directly related to the enhancement of antioxidant enzyme activity, which reflects the improvement of ROS scavenging efficiency in plants [33]. It can be seen that *AmCML24* may enhance the physiological tolerance of plants to saline–alkali and drought stresses by coordinately regulating the osmotic balance and redox homeostasis of plants, and this regulatory mode is consistent with the mechanism of action of most plant stress-resistant genes.

### 3.4. Molecular Mechanisms Underlying AmCML24-Mediated Tolerance to Saline–Alkali and Drought Stresses in Plants

In this study, RT-qPCR was employed to detect the transcriptional levels of genes related to saline–alkali and drought stress responses. The detected genes included salt stress-responsive genes (*AtCML8*, *AtSOS1*), alkali stress-responsive genes (*AtMYB44*, *AtSOS1*), and drought stress-responsive genes (*AtHB7*, *AtABF2*), as well as ABA signaling pathway-related genes (*AtNCED3*, *AtABI4*, *AtMYB71*) [34,35,36,37,38,39,40,41,42]. The selection of these genes covered the core pathways of plant responses to abiotic stresses, providing key transcriptional evidence for the subsequent dissection of the regulatory mechanism of *AmCML24*.

Plant responses to abiotic stress are governed by a complex network modulated synergistically through multiple signaling pathways [43]. The SOS pathway represents one of the key signal transduction pathways for plants to cope with salt stress [44]. The pathway is composed of the Na^+^/H^+^ antiporter SOS1, the protein kinase SOS2, and two calcium sensors—SOS3 and SCaBP8 (SOS3-like calcium-binding protein 8) [45]. Under salt stress, SOS3/SCaBP8 first perceives changes in cytosolic Ca^2+^ signals, then recruits and activates the downstream kinase SOS2. The activated SOS2 further phosphorylates SOS1, enhances its Na^+^/H^+^ exchange activity, promotes intracellular Na^+^ efflux, and maintains cellular ion homeostasis, thereby improving plant salt tolerance [46]. Studies have demonstrated that *AtSOS1*, as a core regulatory gene in the SOS pathway, plays a pivotal role in mediating plant adaptation to saline–alkali stress [35], and this gene is also widely recognized as an important molecular marker for evaluating the activation status of the SOS pathway under saline–alkali stress. To verify the involvement of *AmCML24* in SOS pathway regulation, we analyzed the expression pattern of *AtSOS1* in its overexpressing lines specifically under saline–alkali stress. The RT-qPCR results showed that the expression level of *AtSOS1* was significantly upregulated in transgenic lines compared with the WT control, indicating that *AmCML24*, as a calcium sensor, may bind to calcium ions to activate the SOS pathway and thus enhance plant adaptability to saline–alkali stress by regulating ion homeostasis.

Besides the SOS pathway, the ABA signaling pathway also plays a crucial regulatory role in plant responses to multiple abiotic stresses. Numerous studies have confirmed that calcium-binding proteins, as key regulators of stress responses, can participate in the adaptive regulation of plants to abiotic stresses by mediating the transduction of the ABA pathway [47]. Ding H et al. found that *SlCML39*, as an important negative regulator, plays a key role in the high-temperature stress response, and this process is mediated by the ABA signaling pathway. In *Arabidopsis thaliana*, a novel CML gene from rice, *OsMSR2*, confers drought and salt tolerance to plants through an ABA-mediated pathway [22]. In contrast, *CML20*, an endogenous gene of *Arabidopsis thaliana*, affects the drought-resistant phenotype of plants by negatively regulating ABA signal transduction in guard cells [48]. In addition to the CML family, other types of calcium-binding proteins have also been proven to be involved in the regulatory network of the ABA pathway. *AtCaM9* functions in the adaptation of *Arabidopsis thaliana* to salt stress through the ABA signaling pathway [49], while *AtCPK9* exerts a negative regulatory effect on ABA signaling transduction in stomata [50]. Based on the highly conserved functional characteristics of calcium-binding proteins in the ABA signaling pathway-mediated stress response, this study speculates that the *AmCML24* gene derived from Tibetan peach may enhance plant stress adaptability by participating in the regulation of the ABA signaling pathway.

To verify the hypothesis that *AmCML24* participates in ABA pathway regulation, this study analyzed the expression of *AtNCED3*, *AtABI4*, and *AtMYB71*—key regulatory genes in the Arabidopsis ABA signaling pathway—in different plant lines under NaCl, NaHCO_3_, and mannitol stresses. Experimental data showed that, compared with the WT and mutant lines, these ABA-related genes were significantly upregulated in *AmCML24*-overexpressing transgenic Arabidopsis, directly confirming that *AmCML24* may regulate plant responses to saline–alkali and drought stresses by activating the expression of key node genes in the ABA signaling pathway.

Notably, the ABA signal transduction pathway is widely involved in the response of plants to saline–alkali and drought stress. In further detail, the activation of the ABA signaling pathway relies on the signal recognition and transduction of its receptor proteins: the PYR/PYL/RCAR protein family, which is well recognized as an ABA receptor [51,52]. Under normal growth conditions, the ABA signaling pathway remains inactive; when exposed to drought stress, the endogenous ABA content in plants increases and binds to the receptor proteins PYR/PYL/RCARs. The PYR/PYL/RCAR receptors in plant cells trigger ABA signal transduction through protein phosphorylation. In the presence of ABA, PYR/PYL/RCAR receptors inhibit type 2C protein phosphatases (PP2Cs) by directly binding to ABA [53,54,55]. The inhibition of PP2Cs leads to the activation of SnRK2s (SNF1-related protein kinases 2), which in turn phosphorylate substrate proteins—such as transcription factors, the S-type anion channel SLAC1, and many other targets—thereby inducing stomatal closure in response to ABA [56,57]. While when exposed to saline–alkali stress, the released SnRK2s can achieve self-activation through autophosphorylation and then phosphorylate NADPH oxidase, which increases the content of H_2_O_2_ in the apoplast. This process stimulates the plasma membrane-bound H_2_O_2_ sensor HPCA1, thereby activating the Ca^2+^ channels in guard cells [58]. The increase in cytosolic Ca^2+^ concentration ([Ca^2+^] ^cyt^) can effectively alleviate the damage to plants caused by salt stress.

**Figure 9:**
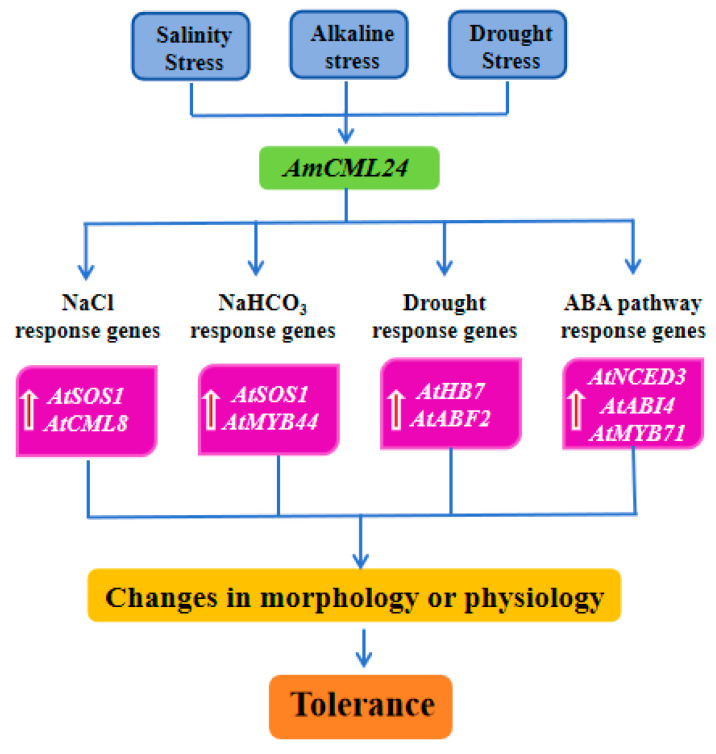
*AmCML24*-mediated response to saline–alkali stress and drought stresses. The arrows in the figure indicate gene upregulation.

In summary, although the molecular mechanism by which *AmCML24*—a member of the calcium-binding protein family—regulates plant stress responses remains to be further elucidated. This study has preliminarily demonstrated that this gene can enhance plant stress tolerance via the coordinated regulation of the SOS ion homeostasis pathway (mediating saline–alkali stress detoxification) and the ABA signaling pathway (mediating responses to drought and saline–alkali stress) with its integrated regulatory network illustrated in Figure 9. Future research can further uncover the specific mechanism of action of *AmCML24* in the crosstalk between the calcium signaling pathway and the SOS/ABA pathways from multiple perspectives, such as protein–protein interaction and subcellular localization.

## 4. Materials and Methods

### 4.1. Plant Materials, Growth Conditions, and Determination of Stress Concentrations

The seeds of Tibetan peach used in this experiment were preserved in our laboratory. The plants were cultivated in a greenhouse under the following conditions: day/night temperature of 28/22 °C, photoperiod of 12 h, light intensity of 250 μmol photons·m^−2^·s^−1^, and relative humidity of 60–70%. Stress treatment assays were performed when the plants reached the three-month-old stage.

The detailed procedures were as follows: First, stress solutions with different concentrations were prepared, and a specific volume of each solution was applied to the plants in individual pots. The fractional irrigation method was adopted; during the first two applications, the treatment solution was ensured to be fully infiltrated and drained. For the third irrigation, the stress solution was retained in the tray to allow continuous absorption by the plants, thus maintaining a constant stress concentration. To ensure the reliability and accuracy of the experimental results, three biological replicates were set up for each treatment group under every stress condition.

The Columbia ecotype of *Arabidopsis thaliana* (Col-0) was preserved in this laboratory. To obtain *AmCML24*-overexpressing plants, the overexpression vector of *AmCML24* was introduced into WT *Arabidopsis thaliana* via an Agrobacterium-mediated transformation method in this study, followed by the acquisition of positive transgenic lines through tolerance screening [59]. The *Arabidopsis thaliana* mutants of *CML24* were purchased from Aruosa Biotechnology Co., Ltd. (Fuzhou, China), with mutant accessions SALK_079293C (corresponding to gene locus AT5G08600) and SALK_058127C (corresponding to gene locus AT3G23150), which are abbreviated as M1 and M2, respectively, in this study. Arabidopsis seedlings were cultivated in a greenhouse with a 16 h light/8 h dark photoperiod, a light intensity of 120 μmol photons·m^−2^·s^−1^, and a constant temperature of 21 °C [60]. All primers involved in this study are available in the Table A1.

The treatment concentrations of NaCl, NaHCO_3_, and mannitol in this study were all determined via pre-experiments. Gradient concentrations of the three reagents were used to treat Arabidopsis seedlings and yeast cells, and the concentrations selected for the formal experiment were those that induced similar levels of growth inhibition without causing lethal damage to the non-transgenic control materials.

### 4.2. RNA Extraction, cDNA Synthesis, and RT-qPCR Analysis

Frozen leaf samples (0.5 g per replicate) were ground into fine powder in liquid nitrogen using a pre-chilled mortar and pestle. Total RNA was isolated from the powder using the Plant RNA Kit (OMEGA, Norcross, GA, USA, Cat. No. R6827-01) following the manufacturer’s protocol; 2 μm of total RNA was used for reverse transcription with ToloScript RT SelectMix for qPCR (Tolo Biotech Co., Ltd., Beijing, China, Cat. No. 22102-01). The synthesized cDNA was diluted 10-fold with RNase-free water and stored at −20 °C for subsequent RT-qPCR analysis. RT-qPCR reactions were performed using 2 × Q3 SYBR qPCR Master Mix (Tolo Biotech Co., Ltd., Beijing, China, Cat. No. 22204); and the relative expression levels of transcripts were calculated using the 2^−ΔΔCt^ method [61], with the 18S rRNA gene serving as the internal reference [62]. All RT-qPCR reactions were performed with three biological replicates and three technical replicates to ensure the reliability of results. Data were statistically analyzed using analysis of variance (ANOVA), and the results were expressed as mean ± standard deviation (SD). Differences between means were compared using Duncan’s multiple range test.

### 4.3. Subcellular Localization Analysis of Transiently Expressed Fusion Proteins

The coding sequence of AmCML24 was fused in frame to the amino terminus of the enhanced green fluorescent protein (GFP) coding sequence, following the method described by (Li et al., 2022) [63]. As a control, GFP transcribed from the CaMV 35S promoter (35S::GFP) was used. The constructs were introduced into epidermal cells of onion bulb mesophyll via particle bombardment. The recombinant vectors described above were transformed into onion epidermal cells via particle bombardment, and the detailed experimental procedure was as follows: A total of 50 μL of 60 mg/mL gold powder suspension was transferred into a 1.5 mL EP tube, followed by sequential addition of 10 μL recombinant plasmid, 50 μL CaCl_2_ (0.25 M), and 20 μL freshly prepared spermidine (0.1 M) protected from light. The mixture was vortexed thoroughly and incubated on ice for 10 min. After centrifugation at 10,000 rpm for 10 s, the supernatant was discarded. The resulting pellet was washed twice with 140 μL of 70% ethanol, and finally resuspended in 60 μL of absolute ethanol. Subsequently, 10 μL of the aforementioned gold powder suspension was used to bombard onion epidermal cells that had been pre-cultured in the dark on 1/2 MS solid medium for 24 h. After bombardment, the cells were further cultured in the dark for 24 h and then sectioned for observation under a fluorescence microscope (ZEISS Axio Imager A2(Carl Zeiss AG, Oberkochen, Germany)). The parameter settings for microscopic observation were as follows: the excitation wavelength of GFP was 488 nm with an emission wavelength range of 500–550 nm; the excitation wavelength of DAPI was 358 nm with an emission wavelength of 461 nm. Three biological replicates were selected, and five fields of view were captured for each replicate to ensure the reliability of the observation results.

### 4.4. Stress Treatment Assays of Recombinant Yeast Strains in Liquid Medium

The full-length coding region of *AmCML24* was fused into the yeast expression vector pYES2. Yeast transformation was performed using the PEG/LiAc method, with reference to the protocol described by (Gietz and Schiestl, 2007) [64]. The empty vector strain pYES2 was used as the control. The recombinant yeast strain pYES2-*AmCML24* and the empty vector yeast strain pYES2 were activated and cultured in YPD liquid medium at 28 °C for 48 h. Subsequently, stress treatment assays were performed in liquid medium following these steps: first, the two aforementioned strains were activated and cultured in YPD liquid medium at 28 °C for 24 h, and then the cell density of both yeast cultures was adjusted to an OD_600_ value of 0.2; next, 1 mL of the culture from each strain was collected and centrifuged to harvest the cells, which were then resuspended in 1 mL of YPD liquid medium supplemented with different stress agents, and the resuspended cultures were incubated at 28 °C with shaking; finally, samples were taken at 3 h, 6 h, and 9 h post-incubation, and the OD_600_ values of the cultures were measured. Three biological replicates were set up for each treatment.

### 4.5. Assays for Seed Germination Rate and Seedling Root Length Statistics

In the determination of Arabidopsis growth characteristics, for the germination rate statistics experiment, seeds of WT, T3-generation *AmCML24*-overexpressing, and mutant lines were first surface-sterilized with 70% ethanol and vernalized in the dark at 4 °C for 48 h. They were then aseptically sown on 1/2 MS medium containing gradient stresses: salt stress (0, 50, 100, 125 mM NaCl), alkali stress (0, 4, 6, 8 mM NaHCO_3_), and drought stress (0, 50, 100, 200 mM mannitol). Each treatment was set with 3 biological replicates (n = 60 seeds per replicate), and the seeds were cultured in an incubator at 22 °C with a 16 h light/8 h dark photoperiod. Starting from 24 h after treatment, germination events (characterized by radicle breaking through the seed coat) were recorded every 24 h, with continuous monitoring for 120 h. For the root length statistics experiment, sterilized and vernalized seeds were sown on vertical plates of 1/2 MS basic medium and cultured until the cotyledons were fully expanded (approximately 7 days). Seedlings with consistent primary root lengths were selected and transferred to 1/2 MS treatment plates with the same gradient stresses mentioned above. Each line was set with 3 biological replicates; after 7 days of treatment, root system images were collected, and root length was quantitatively analyzed using ImageJ version 1.54r software.

### 4.6. Abiotic Stress Treatment and Sample Preparation of Arabidopsis thaliana Seedlings

In the abiotic stress treatment experiment, based on the phenotypic differences in germination rate and root length observed in the previous stage, stress concentrations for whole-plant level treatment were determined: salt stress (200 mM NaCl), alkali stress (100 mM NaHCO_3_), and drought stress (150 mM mannitol). Plump seeds with uniform size from WT, T3 generation *AmCML24*-overexpressing lines, and *AmCML24* mutant lines were selected, vernalized at 4 °C for 3 days, and then sown in nutrient soil with a peat/vermiculite ratio of 3:1. The seedlings were cultured under a 16 h light/8 h dark photoperiod for 21 days until the rosette leaves were fully expanded and the seedlings showed consistent growth vigor. Three biological replicates were set for each line. The stress treatment was conducted via the root irrigation method as follows: 50 mL of stress solution with corresponding concentration was slowly irrigated to the root zone of each seedling to ensure that the solution fully permeated the soil around the roots. The stress treatment lasted for 7 days, during which the same volume of stress solution or distilled water was supplemented daily at a fixed time to maintain a stable stress intensity. After the stress treatment, whole-plant phenotypic photography was performed on the seedlings of each treatment group. Subsequently, uniformly grown seedlings were selected from each group, functional leaves at the same position were excised, quickly frozen in liquid nitrogen, and stored at −80 °C for the subsequent determination of physiological indices and gene expression analysis.

### 4.7. Determination of Physiological Indices

The methods for determining physiological indices were as follows: 0.5 g of leaf tissue from each seedling was weighed out from the −80 °C refrigerator and rapidly ground into a homogenate under ice bath conditions; the homogenate was then transferred to a 1.5 mL centrifuge tube and centrifuged at 12,000 rpm for 20 min at 4 °C. The supernatant was collected as crude enzyme extract/test sample and stored at −20 °C in a refrigerator for subsequent use. Standardized methods were used to detect relevant indices of stressed plants. Soluble protein content was determined by the Bradford method [65]; MDA content was measured by the thiobarbituric acid method [66]; proline content was determined using the acidic ninhydrin method [67]; CAT activity was measured by the ultraviolet absorption method [68]; POD activity was determined via the guaiacol method [69]; and SOD activity was measured using the NBT method [70]. For all index determinations, 3 independent plants were selected as biological replicates for each treatment group, and 3 technical replicates were set up for each biological replicate. The statistical significance of the data was analyzed using one-way analysis of variance (one-way ANOVA), followed by Dunnett’s multiple comparisons test to compare the differences among the transgenic lines, mutant lines, and WT control group.

## 5. Conclusions

In this study, the *AmCML24* was successfully cloned from Tibetan peach, and exhibited a significant response to NaCl, NaHCO_3_, and mannitol stresses. It was localized in the cytoplasm and cell nucleus of cells. Yeast expression experiments demonstrated that overexpression of *AmCML24* significantly enhanced the stress tolerance of yeast strains to the aforementioned stresses. Transgenic Arabidopsis experiments revealed that overexpression of *AmCML24* improved the stress tolerance of Arabidopsis during the germination and root elongation stages. Specifically, it increased the contents of soluble proteins and proline, enhanced the activities of antioxidant enzymes, and reduced the MDA accumulation in transgenic plants. Furthermore, *AmCML24* could synergistically regulate the stress tolerance mechanism by upregulating the expression of stress-responsive genes as well as genes related to the ABA signaling pathway and SOS pathway. Therefore, we speculate that the *AmCML24* gene can indeed significantly improve the saline–alkali and drought tolerance of plants. In conclusion, this study provides insights into further deciphering the molecular mechanism of *AmCML24* in plant responses to abiotic stresses and the construction of its regulatory network, while offering preliminary scientific basis for the genetic improvement, development, and utilization of *Prunus mira* germplasm resources.

## Data Availability

The original contributions presented in this study are included in the article. Further inquiries can be directed to the corresponding author.

## Appendix A

**Table A1:** Primer sequence.

Primer Name	Primer Sequence (5′-3′)
AmCML24-F	ATGTGTCCCTCTGGCCG
AmCML24-R	CTAGCCGACATGATCAACG
pBS-AmCML24-F	TCTAGAATGTGTCCCTCTGG
pBS-AmCML24-R	ACTAGTCTAGCCGACATGATC
q18S-F	AGGTAGCTTCGGGCGCAA
q18S-R	CAGGTTAGCGAAATGCGATAC
qAmCML24-F	GGTTATGGACAAGGACGGGG
qAmCML24-R	CAAGCCAGCATAAGACACGC
pYES2-AmCML24-F	CTCGAGATGTGTCCCTCTGG
pYES2-AmCML24-R	TCTAGAGCCGACATGATCAACG
PBI121-AmCML24-F	TCTAGAATGTGTCCCTCTGGCC
PBI121-AmCML24-R	GTCGACCTAGCCGACATGA
BP	ATTTTGCCGATTTCGGAAC
M1-LP	AGCCTCTTCTTTAGCCATTGC
M1-RP	TTCAACCAAAGGATGAAAACG
M2-LP	CTAAAACCAACGGCTCCTACC
M2-RP	AACTTCACGACCAAGCTCATG
qAtCML8-F	TGCGGAGGAAGAGCTGAAAG
qAtCML8-R	GACTTGGCCATCACCATCCA
qAtSOS1-F	GGTTGCTCTTCCTCCTGCAT
qAtSOS1-R	AAGTTGGATGCAGCGAGTGA
qAtMYB44-F	TCGGGGAAATCGTGTCGTTT
qAtMYB44-R	CTTGAGCGTCGAGTTCCAGT
qAtSOS1-F	GGTTGCTCTTCCTCCTGCAT
qAtSOS1-R	AAGTTGGATGCAGCGAGTGA
qAtHB7-F	GAACAAGAGGGCTCGTTGGA
qAtHB7-R	TCTTCTTTTGCGTCGCCTCT
qAtABF2-F	CAGCCTTCTCCACGAGTTGT
qAtABF2-R	TGATTGGCTGTTGCTGTTGC
qAtNCED3-F	TGATTGGCTGTTGCTGTTGC
qAtNCED3-R	CTGCTTCGCCAAATCATCGG
qAtABI4-F	TCCAAGTTCCGTTACCGTGG
qAtABI4-R	AAGTTGAGCTGAGCACGTGA
qAtMYB71-F	GAGTTTGTGGGGAGGGATGG
qAtMYB71-R	CAACAGAGTTCCATCGCCCT
